# A systematic review of individual and ecological determinants of maternal mortality in the world based on the income level of countries

**DOI:** 10.1186/s12889-022-14686-5

**Published:** 2022-12-15

**Authors:** Maryam Tajvar, Alireza Hajizadeh, Rostam Zalvand

**Affiliations:** Department of Health Management and Economics, School of Public Health, Tehran University of Medical Sciences, Tehran, Iran

**Keywords:** Maternal mortality, Income level, Countries, Determinants, Systematic review

## Abstract

**Background:**

This systematic review was conducted to map the literature on all the existing evidence regarding individual and ecological determinants of maternal mortality in the world and to classify them based on the income level of countries. Such a systematic review had not been conducted before.

**Methods:**

We conducted an electronic search for primary and review articles using “Maternal Mortality” and “Determinant” as keywords or MeSH terms in their Title or Abstract, indexed in Scopus, PubMed, and Google with no time or geographical limitation and also hand searching was performed for most relevant journals. STROBE and Glasgow university critical appraisal checklists were used for quality assessment of the included studies. Data of the determinants were extracted and classified into individual or ecological categories based on income level of the countries according to World Bank classification.

**Results:**

In this review, 109 original studies and 12 review articles from 33 countries or at global level met the inclusion criteria. Most studies were published after 2013. Most literature studied determinants of low and lower-middle-income countries. The most important individual determinants in low and lower-middle-income countries were location of birth, maternal education, any delays in health services seeking, prenatal care and skilled birth attendance. Household-related determinants in low-income countries included improved water source and sanitation system, region of residence, house condition, wealth of household, and husband education. Additionally, ecological determinants including human resources, access to medical equipment and facilities, total fertility rate, health financing system, country income, poverty rate, governance, education, employment, social protection, gender inequality, and human development index were found to be important contributors in maternal mortality. A few factors were more important in higher-income countries than lower-income countries including parity, IVF births, older mothers, and type of delivery.

**Conclusion:**

A comprehensive list of factors associated with maternal death was gathered through this systematic review, most of which were related to lower-income countries. It seems that the income level of the countries makes a significant difference in determinants of maternal mortality in the world.

## Introduction

Maternal mortality is an important public health indicator, reflecting not only poor health condition of mothers and poor quality of health care services, but also in macro levels, it indicates poor economic, social, cultural, and political status in a community and also poor status of women in their society [1–3]. Nowadays, improving maternal health is the priority of most communities and organizations, including the World Health Organization (WHO) [4]. The fifth goal of the eight Millennium Development Goals (MDGs) focused on the reduction of maternal mortality by 75% from 1990 to 2015. Despite intense political attention of the countries toward the MDG5, most of them are still far from achieving their MDG5 targets [3]. The global estimates indicate that 295,000 (UI 279,000 to 340,000) maternal deaths occurred in 2017; 35% lower than 2000 when maternal deaths were estimated at 451,000 (UI 431,000 to 485,000) [5]. Thus, another target for maternal mortality reduction was set by the WHO to reach a global maternal mortality ratio (MMR) below 70 by 2030, which requires reducing global MMR by an average of 7.5% each year from 2015 to 2030. This means that to achieve this goal, maternal mortality must be reduced by more than three times the 2.3% annual rate of reduction observed globally between 1990 and 2015, which would be a difficult task for many countries [6].

Based on the existing evidence, there are significant variations in MMR of countries based on their development and income level [5, 7]. Annually, half a million women die from pregnancy-related deaths, of which 99% happen in developing countries while most of them are preventable [8]. This variation is portrayed by WHO; while MMR has never been passed over 115 cases in Europe, America, and Western Pacific regions since 1990, it has always been over 500 cases in Africa [9].

In order to plan effectively and progress systematically in the reduction of MMR, having a broad knowledge about determinants of maternal mortality is an unavoidable need for policymakers and researchers. These determinants may distantly include socio-economic and cultural factors or intermediately include health status, reproductive status, access to health services, and health care behavior factors, or approximately include biological causes of death [10]. Causes of maternal death, based on the International Classification of Diseases-Maternal Mortality ( ICD-MM), are classified to direct (e.g., pregnancies with abortive outcome) or indirect (e.g., cardiac disease) causes in six groups by WHO [4, 11], with no diversity among them.

The existing review studies on determinants of maternal mortality are generally old, or are non-systematic reviews or only reviewed selected determinants [6, 10, 12]. Recently, only a few studies reviewed the determinants systematically, such as the study of Yakubu et al. [13], but only focused on some micro determinants or other study on limited number of determinants [14, 15]. Given that maternal death is a multidisciplinary phenomenon and different factors are involved, we tried to comprehensively review and overview all determinants, with no limitation, in high quality studies which would be of higher applicability for policymakers and researchers. The aim of this study was to conduct a systematic review on individual and ecological determinants of maternal mortality in the world and to classify them based on the income level of countries. In addition to summarizing distant and intermediate determinants of death in individual or ecological level, we also tried to answer this question that how these determinants might vary among mothers living in countries with various development level.

## Methods

### Inclusion criteria

All types of studies, except descriptive studies, that aimed to identify determinants of maternal mortality were included. In these studies the relationship of possible determinants with maternal mortality was statistically tested. Studies that only focused on the cause of maternal mortality as an approximate determinant, published in non-English journals, or when their full-texts were unavailable were excluded. We considered WHO definition for maternal mortality as “death of a woman while pregnant or beyond 42 weeks of gestation, irrespective of duration and site of pregnancy, due to any cause related to pregnancy or its management, but not due to accidental causes” [9]. No time limit was considered in searches.

### Search strategy

To identify relevant studies, an electronic literature search was first conducted on “PubMed” and “Scopus” databases. Then, some of the most relevant journals were searched specifically (Table 1). Additionally, references of included articles were screened.

**Table 1:** Sources and search terms and the number of studies identified in each (last searched in 5^th^ August 2021)

Searching	Sources	Database /journals	Document types	Key words and Meshes	Publication identified	Final included
**Electronic Searching**	PubMed	Database	All types	(“Maternal Mortality”[MeSH]) AND determinant* [Title/Abstract]	50	52
			Review	(“Maternal Mortality”[Title/Abstract]) AND determinant* [Title/Abstract]	42	
	Scopus	Database	Original	(“Maternal Mortality”) AND determinant*	723	
			Review	(“Maternal Mortality”) AND determinant*	87	
	BMC public health	Journal	All types	(“Maternal Mortality”) AND determinant*	204	23
	Maternal and child nutrition	Journal	All types	(“Maternal Mortality”[Abstract]) AND determinant* [Abstract]	83	
	Maternal and Child Health Journal	Journal	All types	(“Maternal Mortality”) AND determinant*	91	
	BMC Pregnancy and Childbirth	Journal	All types	(“Maternal Mortality”) AND determinant*	397	
	PLoS (One/ Medicine/ Neglected Tropical Diseases/ Pathogens/ Genetics/ Computational Biology)	Journal	All types	(“Maternal Mortality”[Title]) AND determinant* [Title]	126	
**Hand Searching**	Bibliography of the included papers	-	-	-	1855	30
	Google	-	-	-	-	16
**Total**			-	-	3658	121

Except for PubMed database in which “Maternal Mortality” was searched using MeSH term, this term was searched just as a keyword in the rest of search procedure (Table 1). All articles retrieved from electronic databases and other studies were entered into EndNote X8 for further selection.

### Study selection

**Fig. 1:**
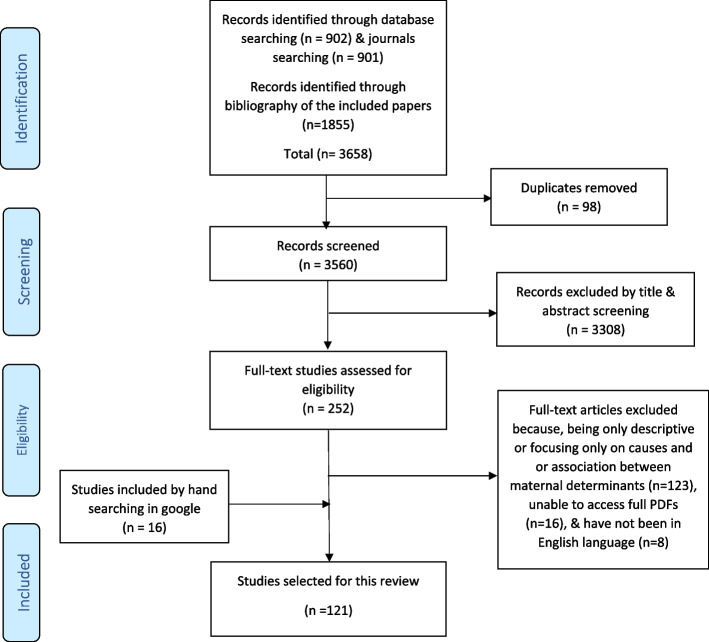
PRISMA flow diagram for systematic review

The process of identification and selection of studies is shown in Fig. 1. Initially, 902 and 901 studies were found through electronic searching in databases and journals, respectively. Furthermore, 1855 records were identified based on the bibliography of the included studies. Duplicate studies (*n* = 98) were removed. Then, screening of titles and abstracts was done by the researchers and studies not relevant were excluded (*n* = 3308). Then, the assessment of the full text of articles was done for eligibility of 252 studies. Based on the exclusion criteria, 123 studies were excluded due to being only descriptive and not reporting statistical results of relationship between independent variables and maternal mortality (except for review articles), or only focusing on biological causes of maternal deaths, or focusing on the relationship between determinants rather than their relationship with mortality. 16 studies were also excluded as their full-text were not found [16–31] and eight others were not in English [32–39]. In addition, 16 relevant articles were added through searching on Google. Finally, 121 relevant studies obtained from electronic databases, bibliographies, and hand searching were selected based on the inclusion criteria (Table 1 and Fig. 1). No study was excluded after quality assessment. Two reviewers (R.Z. and A.H.) performed the literature searching, checking the inclusion and exclusion criteria and selection of studies and finally data extraction, all phases independently. To reach consensus in the case of variations, in all stages of this review, the reviewers met frequently for discussion, and in the case of lasting disagreement, the third reviewer (M.T.), has been intervened.

### Quality assessment

We used the STROBE checklist for original studies and also for ecological studies. It is designed for quality assessment of observational studies especially cohort, case–control, or cross-sectional studies based on 22 items [40]. There were some limitations in quality assessment of the included studies; i.e., some items of this checklist related to participants (item 6), or sample sizes (item 10), or reasons for non-participation (item 13) were not reported in ecological studies that used MMR as a dependent variable. Considering those items as “can’t tell”, the quality scores of those studies decreased. Other items such as clearly defining potential confounders in item 7, reporting potential sources of bias in item 9, way of handling quantitative variables in item 11, and reporting statistical methods used to control confounders or missing data in item 12 of the STROBE checklist were the main limitations of the selected studies, which were not often reported in their methodology. In some included studies, their limitations were not mentioned in the discussion. Each question in this checklist had one score, so the highest quality of the article would obtain score 22.

We used the critical appraisal checklist from Glasgow University [41] for the quality assessment of review articles. As shown in Table 3, except for two studies containing nine review articles, the quality assessment was not performed for the rest of them, and their results were not reported precisely because determinants of maternal mortality were not based on the confidence interval. It is worth noting that the quality of the studies was independently assessed by two researchers in different places. Then, discrepancies were resolved by participation of a third researcher to reduce the risk of bias.

### Data extraction

We extracted the required data from the included review articles and original articles using a purposefully designed data extraction form. Year of publication, number of maternal deaths, time span of the study, setting, design, and methods, and maternal mortality determinants were extracted from the included studies as shown in the Tables 2 and 3.

**Table 2:** Summary of characteristics and findings of original studies (Chronologically ordered)

**Date of pub/ Ref**	**N of deaths,** **setting & date**	**Income**	**Design**	**Key findings**		Quality (0–22)
				**Factors Associated**	**Factors Not associated**	
1 2021 [42]	*N* = 43 Indonesia 2017–2019	LM	Case–control	Education, parity, age, family income	Occupation	20
2 2021 [43]	*N* = - China 2013–2018	LM	Ecological	GDP per capita, rate of delivery in maternal and child health hospital, rate of cesarean section, rate of low birth weight	-	19
3 2021 [44]	*N* = - India 1997–2017	L	Ecological	Place of residence, education, region of residence, wealth index, health insurance coverage, caste	Religion, number of members in the household	21
4 2021 [45]	*N* = - 54 African countries 2006–2018	L	Ecological	Gender inequality index, current health expenditure as a percentage of GDP, density of skilled health professional, type of delivery, lifestyle, gender inequities, the proportion of skilled birth attendant	-	17
5 2020 [46]	*N* = 47 Pakistan 2013–2017	L	XS	Poverty, prenatal care, education, long distance, delay in acquiring blood/blood products, delay in surgery	-	16
6 2020 [47]	*N* = 367 Cameron 2004–2011	L	Ecological	Community education, community wealth, age, parity, social autonomy, domestic violence, distance to facility, media exposure, contraception type, education, religion, ethnicity, type of residence, wealth index, water quality		18
7 2020 [48]	*N* = 33 Indonesia, 2015–2017	LM	XS	Education, age, occupation, maternity period, parity, distance from health center	-	20
8 2020 [49]	*N* = 14,892 South Africa 2007–2015	L	Ecological	Age, education, marital status, occupation, place of death	-	19
9 2020 [50]	*N* = - China 2004–2016	LM	Longitudinal	Level of development of provinces, proportion of illiteracy, average annual household consumption, proportion of college educated, maternity health insurance coverage, premarital check-up, maternal health profiles creation rate, coverage of maternal systematic management, prenatal care, postpartum visit rate, hospital delivery rate, delivery attended by professionals rate	Public budget in health sector per capita, density of specialized maternal and child health hospitals, density of health providers in specialized maternal and child health hospitals	22
10 2019 [51]	*N* = 8075 Indonesian (rural) 2010 census	LM	XS	Age, education, health service access, number of midwives working in the village, doctors working at health center	Education of the household head, spouse employment, midwives working at health centers	19
11 2019 [52]	*N* = - Brazil 2013	UM	Ecological	Human Development Index (HDI), proportion of family health care strategy implementation, caesarean rate	-	17
12 2019 [53]	*N* = - USA 2007–2015	H	Ecological	Rural residents, type of health insurance, type of delivery, racism, poverty	-	19
13 2019 [54]	*N* = 810 Nigeria 2008–2013	L	XS	Education, community wealth, media exposure, religion, distance to facility, contraception type, ethnicity, type of residence, wealth index, water quality, age	-	18
14 2019 [55]	*N* = - India 2016–2017	L	Case control	Age, parity, provider of labor	Place of death/delivery, time of occurrence	19
15 2019 [56]	*N* = 150 India 2013–2014	L	XS	Occupation, husband’s occupation, accesses to health services	-	18
16 2019 [57]	*N* = 59 Ethiopia 2014–2017	L	Case–control	Obstetric, husbands education, antenatal care, home delivery, place of residence, parity	Place of death, state of pregnancy at time of death	20
17 2018 [58]	*N* = 100 Zambia 2010–2015	L	Case–control	Presence or absence of risk condition, antenatal care, presence of complications, any delay to seek health care, delay type, Parity, gestational age, care time, sought traditional health care, marital status, education	**-**	20
18 2018 [59]	*N* = - USA 1997–2012	H	Ecological	Education, African American race, prenatal care visits, state median income, ethnicity, un insurance rates	Age and cesarean-section rates	20
19 2018 [60]	*N* = 312 South African 2000–2014	LM	Longitudinal	Wealth index, source of drinking water, education, period of death, area and number of deliveries, period of death 2000–2006	Age, distance to the nearest clinic	18
20. 2018 [61]	*n* = 91 Nigeria 2014	LM	XS	Type of facility, booking status for antenatal care, referral, Parity & number of early pregnancy loss	Occupation, Place of delivery, complication in index pregnancy & Mode of delivery	21
21. 2017 [62]	*n* = 16 Ethiopia 2011–2014	L	XS	Place of delivery, occurrence of hypo volume shock, & postoperative severe anemia	-	21
22. 2017 [63]	*n* = 1103 Nigeria 2013	LM	XS	Wealth index, residence, community women’s education, autonomy, spousal educational difference, highest educational attainment, age, religion, & attitudes towards domestic violence	Economic empowerment	21
23. 2017 [64]	- China 2002–2014	UM	Ecological	Social and economic environment (GDP per capita, rate of urbanization and highway per land, the average travelling time to the nearest hospital), Health human resources, health infrastructure, & maternal health care service	-	20
24. 2017 [65]	*n* = 16 China 2011	UM	XS	Mode of delivery including operative vaginal delivery, non-indicated intrapartum, indicated antepartum, & indicated intrapartum	Non-Indicated antepartum as a mode of delivery	20
25. 2017 [66]	*n* = 39 Indonesia 2017	LM	Case–control	Late decision making, complication, standard ANC visit, high risk pregnancy, & high educational level	Late transportation providing, employment status & Late handling in medical facilities	19
26. 2017 [67]	- Ecuador 2014	UM	Ecological	GDP, percentage of households with children who do not attend school, percentage of households with electricity, percentage of indigenous population, & total fertility rate	Poverty with unsatisfied basic needs, Average household income, Percentage of households with inadequate services, & Average persons per bedroom	18
27. 2017 [68]	- low-income countries 2012- 2015	L	Ecological	Total fertility rate, skilled birth attendance	Corruption index, GDP per capita, & empowerment measures	17
28. 2017 [69]	*N* = 37 Indonesia 2010–2012	LM	XS	Education, Referral status, location of death, history of maternal systemic illness, Type of labor (e.g. spontaneous), pregnancy complications, & puerperium complications	Labor complications, maternal age, parity, pregnancy interval, total antenatal care, first labor assistance, spouse occupation & residence	16
29. 2017 [70]	*n* = 360 Nigeria -	LM	XS	Place of consultation, who pays the treatment costs, awareness of pregnancy complications and knowledge of the place of antenatal care treatment	-	15
30. 2016 [71]	*n* = 73 Brazil 2009–2012	UM	Case–control	Mode of delivery, premature delivery, gravidity, education, and age	Skin color, type of hospital, & region of residence	21
31. 2016 [72]	*N* = 34 Iran 2009—2013	UM	XS	Gravidity	Residence, type of birth, birth attendant, and mother’s age and place of birth	19
32. 2016 [73]	- LMICs 1990 -2010	-	Ecological	Gender equality, infrastructure, education, water and sanitation, GDP per capita, fertility, Immunization, & health services delivery	-	19
33. 2016 [74]	*n* = 89 Cameroon 2011–2014	LM	Case–control	Inadequate antenatal care, pre-existing co-morbidities, place of delivery, healthcare provider qualification, and delays in arrival at health facilities	Age, marital status, occupation, mothers education, religion, residence, way of transport, & parity	19
34. 2016 [75]	328 Turkey 2002–2013	UM	Longitudinal study	Cesarean delivery	-	19
35. 2016 [76]	- East Mediterranean 2004–2011	-	Ecological	GDP Per Capita, health expenditure as % of GDP, Literacy rate among Female adults aged 15 + , Birth attended by skilled health providers, Primary Healthcare units and Centers, & Urbanization Rate	Total Fertility Rate (TFR)	17
36. 2016 [77]	*n* = 313 Uganda 2011	L	XS	Mothers education, age, region of residence, number of visits	-	14
37. 2015 [78]	*n* = 62 Ethiopia 2012–2013	L	Case–control	Member of women’s development army, husband’s involvement score, ever used contraceptives before the last pregnancy, & any previous medical illnesses,	Involved in health care decision making, Number of antenatal care visits, & Previous pregnancy-related complications	20
38. 2015 [79]	- Global 2000–2010	-	Ecological	% of access to water, % of access to sanitation systems, birth attention by a healthcare professional, having corrupt government, total Health expenditure (Q2 vs Q1), & fertility rate (Q4 vs Q1)	total Health expenditure (Q3 & Q4 vs Q1), Human resource & fertility rate (Q3 & Q2 vs Q1)	20
39. 2015 [80]	*n* = 135 UK 2009–2012	H	Case–control	Inadequate use of antenatal care, substance misuse, medical comorbidities, previous pregnancy problems, & Indian ethnicity	Age, parity, BMI, employment status, & result of the birth	20
40. 2015 [81]	*n* = 28 Ghana 2013	LM	XS	Age, residence, parity, education, religion, antenatal care, lack of communication between facilities, lack of equipment, inadequate treatment, patient & facility delay	Occupation of mothers	19
41. 2015 [82]	*n* = 402 LMICs 2010–2013	-	Longitudinal	Age, parity, Birth attendant, education, lack of antenatal care, & caesarean section delivery	-	19
42. 2015 [83]	- Global 2010	-	Ecological	Private sector and trade, governance, education, employment and social protection, economic policy and debt, health- service expenditure, environment- agriculture and Production, education-efficiency, &private sector- private infrastructure	-	19
43. 2015 [84]	*n* = 51 Ethiopia 2012–2013	L	XS	Population density	-	18
44. 2015 [85]	*n* = 35 Indonesia 2013–2014	LM	Case–control	High risk of health status (nutritional status, anemia, disease history and previous pregnancy complications)	-	17
45. 2015 [86]	*n* = 13 Ethiopia 2014	L	XS	Occupation, ethnicity, age, and religion	Marital and educational status	16
46. 2015 [87]	- China 1996–2009	LM	Ecological	Increased hospital delivery rate, decreased male illiteracy rate, increased GDP	-	15
47. 2014 [88]	*n* = 109 China 2010–2011	UM	Case–control	Maternal age, education, gravidity, parity, marital status, having health insurance, antenatal care, family income, mode of delivery, not registered in healthcare system, no decision on ANC attendance made by woman, not received follow-up reminders to attend ANC, not knowing the recommendation to deliver in hospital, not knowing the danger signs of delivery, not knowing to register the pregnancy and to attend antenatal care (ANC), & unplanned pregnancy	Complications during pregnancy	21
48. 2014 [89]	- European Union 1981–2010	-	Ecological	Government healthcare expenditure	-	20
49 2014 [90]	*n* = 209 Malawi 2001–2002	L	Case–control	Condition on admission, outcome of birth, number of days in hospital, transfusion, evacuation, & birth weight	High parity	20
50. 2014 [91]	*n* = 140 Brazil 2009 -2010	UM	XS	Delay in seeking health services, quality of medical care (including absence of blood products, lack of medication, difficulty in communicating between hospital and regulatory center, lack of trained staff, difficulty in monitoring, improper patient management, and delays in starting treatment, diagnosis and transfer)	Refuse to treatment, Unsafe abortion, health service accessibility	20
51. 2014 [92]	*n* = 150 Kenya 2004–2011	L	Case–control	Age, education, history of underlying medical conditions, contraceptives, gravidity, mode of delivery, birth attendant, pregnancy stage, number of ANC visits, comorbid complication, referral, & pulse	-	19
52. 2014 [93]	*N* = 37 Iran 2006 -2014	LM	XS	Maternal age, gravidity and preterm of gestational age	Type of delivery and birth attendant	18
53. 2014 [94]	*n* = 226 Brazil 2000–2002	UM	Case–control	Type of hospital deliveries, mode of delivery, & residence	Age, education	18
54. 2014 [95]	*n* = 35 Indonesia 2013–2014	LM	Case–control	Age at marriage, education, occupation, gender and culture (e.g. involving in decision-making)	-	17
55. 2014 [96]	- LMICs 1990–2010	-	Ecological	GDP per capita, doctors per 100 people, prenatal care, access to sanitation system, total fertility rate, female total years schooling, total years schooling in both sexes, Power consumption per capita, roads paved, & Published scientific papers annually	Poverty, government effectiveness index, skilled birth attendance, measles immunization, HIV prevalence, health expenditure per capita, out-of-pocket health spending, access to clean water, contraceptive prevalence, percentage of women in parliament, female labor force participation	13
56. 2013 [97]	*n* = 486 Global 2010- 2011	-	XS	Marital status, age, schooling, number of previous births, number of previous caesarean sections, onset of labour, & mode of delivery	-	21
57. 2013 [98]	*n* = 62 Ethiopia 2010–2012	L	Case–control	Level of education, residence, distance to nearby hospital, ANC registration history, age of the women, types of birth attendants, length of labor from admission & mode of delivery	-	17
58. 2013 [99]	- Madagascar 1993–2007	L	Panel study	Primary clinics [4], time to the Hospital (hours), population, births (number divided by 1,000), female literacy, female wages (weekly, national currency divided by 1,000), income, poverty gap, & without toilet (number per households)	Doctors (per 1,000 population)	15
59. 2012 [100]	*n* = 7 Sub-Saharan Africa 2010–2011	-	XS	Parity	Maternal age	19
60. 2012 [101]	- Iran 2001–2008	LM	Ecological	Male literacy, unemployment, & female literacy	Proportion of midwives, urban residency	17
61. 2012 [102]	- Chile 1957 to 2007	LM	Time-series	Education, TFR, delivery by skilled attendants, & clean water, sanitary sewer	GDP per capita	16
62. 2012 [103]	1129 China 1996–2009	LM	Longitudinal	Prenatal care, gestational age, pregnancy tendency(wanted/ unwanted), place of death, income, residence & education	-	15
63. 2011 [104]	*n* = 363 Global 2004–2005	-	XS	Maternal age, marital status, parity, government health expenditure, Gravidity, & Education	-	21
64. 2011 [83]	*n* = 328 France 2001–2006	H	Case–control	Residence, maternal age, parity, hospitalization during pregnancy, induced laboar, type of delivery, emergency cesarean, preterm delivery, & multiple birth	Work status, marital status	21
65. 2011 [105]	- Iran 2004–2006	LM	Ecological	Human development index, difference in illiteracy rate in women and men	Gini coefficient	20
66. 2011 [106]	- Global 2001–2008	-	Ecological	Access to improved sanitation and water, corruptive government, health expenditure, & total fertility rate	-	18
67. 2011 [107]	- Nepal 1996–2006	L	Ecological	% Professional delivery care, % hospital delivery, % health facility delivery, % emergency obstetric care facility delivery, % caesarean section delivery, % met need for emergency obstetric care, mean birth order, mean wealth quintile, mean total children born, mean age at first birth, mean hemoglobin, % anemia moderate/severe, % any birth preparation, Human Development Index, Gender Empowerment Measure, General Fertility Rate	GDP	18
68. 2010 [108]	*n* = 47 Africa 2004	L	Case–control	Travel times more than 4 h from hospital	Travel times between 2–4 h from hospital	21
69. 2010 [109]	*n* = 32 India 2003–2004	L	Case–control	Percentage of people under poverty line, mothers age, number of pregnancy, & place of delivery	Education, prenatal care	20
70. 2010 [110]	- high and middle income 1936–2005		Time-series	GDP per capita	-	16
71. 2010 [111]	*n* = 46 India 2003–2006	L	Retrospective cohort	Mode of delivery, parity, age	-	16
72. 2009 [112]	*N* = 339 china 1997	LM	XS	Average number of village doctors, proportion of villages without doctors, percent of minority groups	-	20
73. 2009 [113]	- Ethiopia 1995–2008	L	Time series	GDP per capita, adult literacy rate, total fertility rate, number of doctors per 100,000 population, number of nurse per 100,000 population, hospitals per 100,000 population, skilled birth attendance, postnatal care coverage, prenatal care coverage, & Recurrent Health Expenditure per capita	-	20
74. 2009 [114]	*n* = 148 Spain 1996–2005	H	XS	Maternal age	-	19
75. 2009 [115]	- Sub-Saharan Africa 1997 – 2006	-	Ecological	Infant mortality (deaths per 1000 live births), antenatal care coverage (%), births attended by skilled health personnel, access to an improved water source, adult literacy rate, contraceptive prevalence, ratio of female rate to male rate, primary enrolment rate, primary enrolment rate, female, education index, Gross National Income per capita ($),out-of-pocket expenditure on Health, per-capita government expenditure on health ($)	Access to improved sanitation, public expenditure on health (%GNP), public expenditure on education (%GNP), external resources for Health, government expenditure on Health	17
76. 2009 [116]	*n* = 112 Nigeria 2003–2007	L	XS	Parity, antenatal care, literacy	Maternal age	16
77. 2008 [117]	*n* = 45 Tanzania 1995–1996	L	Case–control	Maternal age, ethnic and religious affiliation, and low formal education of the husbands	-	21
78. 2008 [118]	*n* = 267 France 1996–2001	H	Case–control	Nationality, age, parity, work status, hospitalization during pregnancy	Marital status	21
79. 2008 [119]	*n* = 387 Burkina Faso 2002–2006	L	XS	Antenatal care coverage	Asset index quintile, distance to hospital, distance to health facility, caesarean section rate, institutional birth rate	19
80. 2008 [120]	*n* = 84 Nigeria 2003–2004	L	Case–control	Long labor, grand multi parity, Higher occupational status, No antenatal care	Age, birth interval	15
81. 2007 [121]	*n* = 769 Bangladesh 1976–2005	L	Retrospective cohort	Pregnancy order, maternal age (years), household asset quintile among less/ least poor people, completed years of formal education of mother, service area	Religion, household asset quintile among poorer or poor people	20
82. 2007 [122]	*n* = 70 India 1993–1995	L	Case–control	Age, education of wife, education of husband, type of family, parity, antenatal care receiving, place of delivery, mode of transport used to reach the health facility, complication during pregnancy & distance of the health facility from residence	Caste, age at marriage of women	18
83. 2007 [123]	*n* = 84 Argentina 2002	UM	Case–control	Age, gravidity, Obstetrics-gynecology specialist on 24-h active duty, Availability of essential obstetric care, Length of hospitalization (days), Presence of residency program, & Previous gestations	Education, marital status, cesarean section	18
84. 2007 [124]	- China 1990–2000	L	Time series	modern delivery methods, GDP per capita, illiteracy rate, hospital delivery, clean drinking water	contraceptive use & Prenatal care	15
85. 2006 [125]	- Africa’s countries 1990–2010	-	Ecological	CNP per capita, policy instability	-	19
86. 2006 [126]	*n* = 153 Senegal 1997–2000	L	XS	Previous Caesarean section, prenatal visits, referral for delivery, mode of delivery, transfusion	Age, parity	18
87. 2006 [127]	- Global 2003	-	Ecological	Human development index, Gender-Related Development Index, female literacy rate, combined primary, secondary, and tertiary enrolment ratio, & Infant mortality rates	Gender Empowerment Measure, Total fertility rate, female professional and technical workers, ratio of estimated female to male earned incomes, female political involvement, & women participation in parliament	13
88. 2005 [128]	- US 1994–2001	H	Ecological	Specialist ratio per 10,000 live birth, obstetric/ provider ratio per 10,000 live birth, general obstetric/gynecologist ratio per 10,000 live birth, poverty, Percent mothers without a high school diploma, Percent teenage mothers, & intercept	Color of skin, Percent mothers without adequate PNC	14
89. 2004 [129]	- Global 2004	-	Ecological	Nurse density, doctor density, & Gross national income per person	Female adult literacy, & poverty	20
90. 2004 [130]	- Sub-Saharan Africa 1990–2003	-	Ecological	(GNP) per capita, health expenditure per capita, births attended by skilled health personnel & life expectancy at birth	HIV/AIDS prevalence, Total fertility rate, Political stability	19
91. 2004 [131]	- developing countries 1984	L	Ecological	Poverty, education, type of floor, type of toilet, & source of water	-	16
92. 2003 [132]	*n* = 59 Indonesia 1996–1999	L	Case–control	Residence, employment status, availability of toilet facility, & time of initial visit to antenatal care facilities	Race, religion, travel time to hospital, availability of automobile, access to clean water, Antenatal care, education, facilities, birth interval, & age at first delivery	19
93. 2002 [133]	- Nigeria 1990–1999	L	Ecological	Place of residence and literacy level	poor obstetric history (abortion, stillbirth and premature birth)	19
94. 2002 [134]	*n* = 85 Guinea-Bissau 1990	L	Case–control	distance from the regional hospital, region of residence, Birth interval, Distance to health center, health post in village & result of the birth	Age, parity, & Access to latrines	18
95. 2002 [135]	*n* = 104 Nigeria 1990–1999	L	XS	Age, parity, & Booking status	-	13
96. 2001 [136]	- Global 1998	-	Ecological	Fertility rate, immunization with DPT, Urban population, & female working in industry	-	18
97. 2001 [137]	- Global 1988–1997	-	Ecological	GNP per capita, physician and nurse per 1000 people, female literacy, & skilled birth attendance	-	16
98. 2000 [138]	- Global -		Ecological	Income per capita, health expenditure, antenatal care, receiving trained assistance at childbirth, female gross primary-school enrollment rate, & female gross secondary-school enrollment rate	-	17
99. 1999 [139]	- less developed countries 1970–1990	L	Ecological	education relative to men, age at first marriage, reproductive autonomy, GDP per capita, total fertility rate, economic growth, Contraceptive prevalence, population per doctor, Health attendants, & male to female ratio secondary school enrollment	Foreign calories per capita investment	15
100. 1999 [140]	*n* = 583 Accra-Ghana 1990–1997	L	XS	Vaginal delivery, postpartum timing of death, age, & formal education	Cesarean delivery, instrumental delivery, antepartum timing of death, intrapartum timing of death, residence, marital status, parity	13
101. 1998 [141]	*n* = 261 Pakistan 1991–1993	L	Case–control	Age, Parity, Previous history of fetal loss, access to electricity, accessibility of essential obstetric care, & Number of rooms in the house	Husbands literacy, & birth attendant	20
102. 1998 [142]	*n* = 121 India 1993–1995	L	Case–control	Overall distance travelled from home to appropriate treatment facility, distance of first health service contact, & distance between first heath service contact and appropriate treatment facility, place of delivery, type of attendants, & husbands education	Use of contraceptives, women education, age, & higher economic status	18
103. 1997 [143]	*N* = 218 Pakistan 1989–1992	L	Case–control	Household assets, Distance from nearest hospital, Housing Construction material, Availability of electricity, Water supply, Marital status, Age, Gravidity, & Obstetric History	Education, Husband’s education, Employment status	21
104. 1997 [144]	*n* = 152 Senegal 1986–1987	LM	Case–control	Medical equipment, late referral, no antenatal visits, Any complication during pregnancy, Previous C-section, Never treated previously, & Previously hospitalized	No one available at time of admission, Prenatal visit to a specialized center	18
105. 1997 [145]	- Global 1987—1990	-	Ecological	Human development index including life expectancy, education, & GDP per capita	-	17
106. 1996 [146]	*n* = 1341 England and Wales 1970–1985	H	XS	Age, & place of birth	-	20
107. 1994 [147]	- Global 1979- 1981	-	Ecological	a households without sanitation, total calories consumed, & total fat residual,	fat calories consumed, medical personnel available, no safe water, hospital beds available, literacy rate	17
108. 1994 [148]	*n* = 271 India 1984–1992	L	XS	Gestational age, Level of consciousness, & convulsions	-	16
109. 1993 [149]	159 Zimbabwe 1989–1990	LM	Case–control	Marital status in rural, head of household in rural, catholic religion in urban, still birth, & history of abortion in urban	Marital status, education, guardian family, religion, income, gravidity, parity, result of birth (live birth), wanted pregnancy, history of abortion	17

Strobe-checklist tool was used for quality assessment of original studies

*LMICs* Low- and Middle-Income Countries, *L* Low income, *LM* Lower Middle income, *UM* Upper Middle income, *H* Higher income, *XS* Cross sectional study

**Table 3:** Summary of characteristics and findings of review studies

**Publication date/ Ref**	**Date**	**Type of review**	**Number of included studies**	**Key findings** (factors associated with maternal death)	**Quality of studies**^a^									
					Did the review address a clearly focused issue?	Did the authors look for the appropriate sort of papers?	Do you think the important, relevant studies were included?	Did the reviews authors do enough to assess the quality of the included studies?	If the results of the review have been combined, was it reasonable to do so?	What is the overall result of the review?	How precise are the results?	Can the results be applied to the local population?	Were all important outcomes considered?	Are the benefits worth the harms and costs?
1.2020 [150]	2005–2015	SR	25	Low socio-economic status family, rural and remote areas, teenagers and unmarried women, perceptions related to pregnancy, delivery and death, perception related to family planning, practice related to pregnancy and delivery, gender inequity, perception related to midwife performance and health services	Y	Y	CT	Y	Y	N	Y	CT	Y	Y
2.2019 [151]	-	SR	29	Preterm of gestational age, obesity, quality of care, immune pregnancy ratio, human development index, gender inequality in education, access to improved water source, fertility rate	Y	Y	Y	Y	Y	Y	N	Y	Y	Y
3.2019 [152]	2007–2019	SR	7	Age, parity, domestic violence, inadequate antenatal care, place of delivery, education, healthcare provider qualification, delays in arrival at health facilities, unemployment, socioeconomic status, no antenatal care, having been referred, distance to health facilities, social autonomy, media exposure, family planning, nutrition, religion, ethnicity, wealth index, water quality, sanitation and hygiene	Y	Y	Y	N	Y	Y	Y	N	Y	Y
4. 2018 [13]	since 2000	SR	33	Parity, education, fertility level, maternal age at birth, status of women, income/poverty, access to electricity or access to improved water sources and sanitation	Y	Y	Y	N	Y	Y	Y	Y	CT	Y
5. 2016 [14]	-	SR	62	harmful traditional beliefs and practices; early marriage, high parity and female illiteracy	Y	Y	Y	Y	CT	Y	CT	Y	CT	CT
6. 2014 [15]	Since 1980	SR	14	water and sanitation system	Y	Y	Y	Y	Y	Y	Y	Y	Y	Y
7. 2017 [153]	-	NR	38	Factors including education, sociocultural practices (e.g. criminalization of marriage blow 18, and Promoting women’s rights), social services (e.g. Improved health care access and quality), and health care (access, utilization, and quality), and income or employment	Y	Y	Y	N	Y	Y	N	Y	Y	Y
8. 2015 [154]		NR	-	Economic growth, Poverty, Inequality, Local infrastructure, education, Access to public health information, Fertility rates, Average age of first pregnancies, Urbanization, Women’s empowerment, income, living condition, Affordability of services, health status, Age & age of first pregnancy, birth spacing, previous fetal loss, urban location, and proximity to services at the community or social and individual levels, access to family planning, antenatal support, delivery & post birth care, and quality of services, and demand of this services	Y	Y	Y	N	Y	Y	Y	CT	Y	Y
9. 2006 [155]	1975–2001	NR	9	Mode of delivery may **not** associated with maternal mortality	Y	Y	C	N	CT	Y	N	CT	Y	CT
10. 1998 [12]	in 1996	NR	-	Wife seclusion, Age, universal female illiteracy, harmful traditional medical beliefs and practice, inadequate facilities to deal with obstetric emergencies, a deteriorating economy & policy	Y	N	N	N	Y	Y	N	Y	CT	CT
11. 1992 [10]	in1992	NR	-	Distant determinants (education, occupation, income, social and legal autonomy, wealth and community resources), and intermediate determinants (use of family planning, use of prenatal care, use of modern care for labor and delivery, use of harmful traditional practices, use of illicit) and induced abortion, location of services, range of services available, quality of care, access to information about services, age, parity, marital status, nutritional status, infections and parasitic disease, and prior history of pregnancy complications	Y	Y	N	N	CT	Y	N	Y	CT	CT
12. 1991 [6]	1980–1990	NR	-	Poor sanitation, lack of clean water, poor transportation, lack of medical facilities, malnutrition, poverty, increased work load, frequent pregnancy, low status of women, early marriage, lack of legal abortion, & no acceptance of contraception	Y	Y	CT	N	Y	Y	N	Y	Y	CT

*SR* Systematic Review, *NR* Narrative Review, *Y* Yes, *N* No, *CT* Can’t Tell

^a^Glasgow university critical appraisal checklist [41] was used for quality assessment of these review articles

### Data analysis

The included studies were developed with diverse objectives, used various methods and different statistical techniques, and included participants with different characteristics and were widely distributed among countries. This diversity made formal meta-analysis almost impossible. Some of the included studies were developed with similar objectives, and participants had similar characteristics and used similar methods and measures, but we also classified the determinants of maternal mortality into three main individual, household, and community levels to be higher applicability for policymakers and researchers.

### Classification of the included studies based on income levels of countries

We classified each original study based on low-, lower-middle-, upper-middle-, and high-income countries according to World Bank classification. Some points considered in this classification; for example, if a study in a country was in a specific income group for a some years and then its level changed for next years, we chose the income group that were for a longer period. In the study of Simo ˜es et al. [94], Brazil was in the upper-middle-income group for two years (2000–2001) and then changed as a lower-middle-income country in 2002; thus we considered Brazil in the upper-middle-income group. If a study included different countries in two different income groups, we chose the group with more countries. For example, in the study of Graham et al. [131], among 11 different countries, only two countries were in lower-middle group and the rest were in low-income group; so, we considered it as a low-income country. We also chose the last years of income level for the studies that used the same number of years for two different levels of income. For example, in the study of Taguchi et al. [77], Indonesia was lower-middle-income country for two years (1996 and 1997) and was low-income for the other two years (1998 and 1999), so we considered Indonesia as low-income country. Studies with more than two income groups in different countries were considered as global/regional studies, illustrated in the last column of Table 4.

**Table 4:**
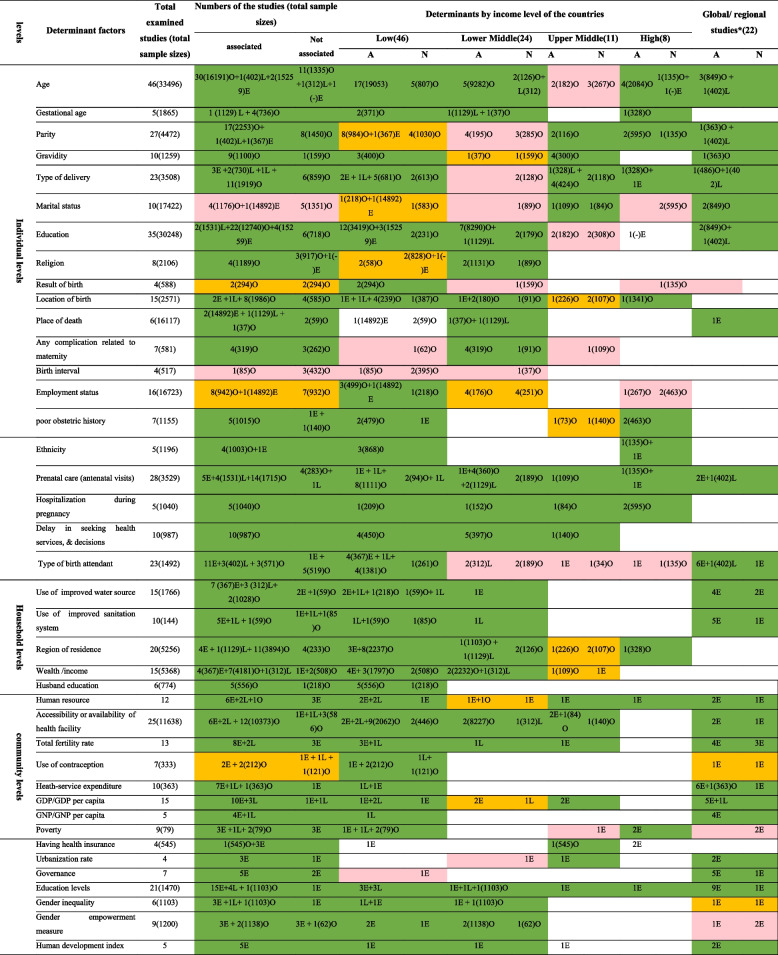
Summary of determinants of maternal mortality according to the Table 2

*A* Associated, *N* Not associated, *O* Observational studies (including case–control, cross sectional & retrospective cohort studies), *L* Longitudinal studies (including prospective cohort, panel and time-series studies), *E* Ecological studies

^a^These studies used more than two types of different income level groups among different countries. The colors shows the association of the determinants based on numbers of the studies, total sample sizes & sometimes quality of the included studies (  There was significant relationship,  there was no consensus &  there was not significant relationship). Strobe-checklist tool were used for quality assessment of these original studies

## Results

The results of this systematic review are structured here in two main sections; first, according to Tables 2 and 3, which are a summary of all included original studies and review articles, respectively, and provide a description of the characteristics of included studies. Then, maternal mortality determinants are reported for review articles and original studies, summarized at individual, household, and community levels by income groups in Table 4.

### Description of the included studies

**Fig. 2:**
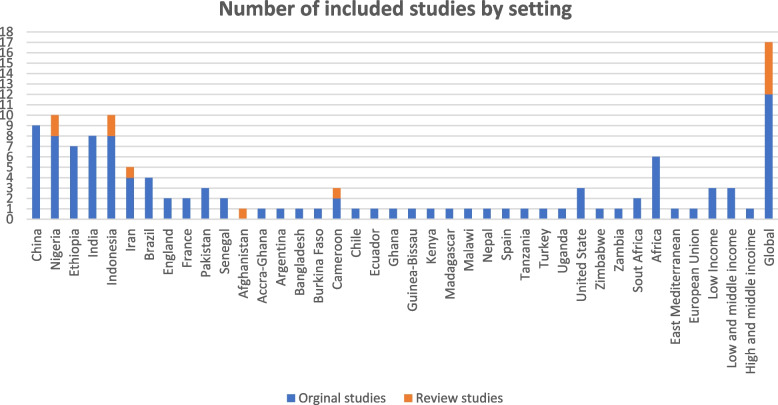
Number of included studies by setting

**Fig. 3:**
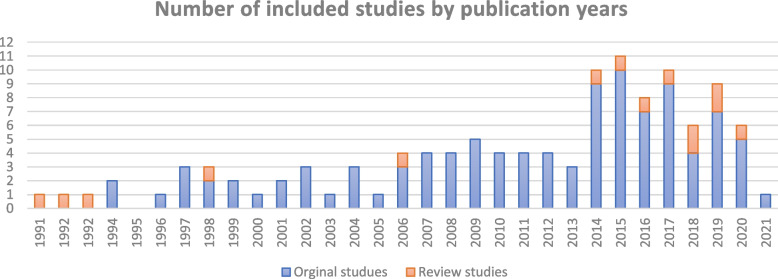
Number of included studies by publication years during 1991–2021

Of 121 included studies, 12 were review articles (Table 3) and the rest were original studies (Table 2) in which 33%, 28%, 27%, 10%, and 2% were ecological, case–control, cross-sectional, longitudinal (including prospective cohort, panel and time-series studies), and cohort studies, respectively. Among the 12 review articles, six of them with a total of 160 studies were conducted systematically and the rest were narrative reviews (Table 3). Most of the literature evaluated the determinants of maternal mortality globally with special focus on low- and lower-middle-income countries. Moreover, the results by countries show that most of the included original studies have been conducted in China, followed by Niagara and Indonesia (details provided in Fig. 2), and most of them were published after 2013 (Fig. 3).

### Determinants of maternal mortality in the world

In this section, first the results of the review articles then the original study’s findings are reported based on determinant levels and income levels of countries.

Based on our review articles, some macro factors such as economic growth, poverty, inequality, improved water and sanitation, education, accessibility of health care services, etc. (details are shown in Table 3), and some micro factors such as age, parity, type of delivery, early marriage, etc. have led to maternal mortality.

### Maternal mortality determinants based on individual, household, and community levels

Determinants at individual level mainly show the health status and reproductive-demographic characteristics of deceased mothers. A summary of the determinants of mothers who died due to pregnancy-related reasons, based on original studies, is described in Table 4. As shown in column three of the table, among demographic factors, maternal age over 34 or below 18 is one of the main factors affecting pregnancy-related deaths, and nearly 70% of 36 examined studies with 7632 aggregate deaths confirmed this association. In addition, higher gravidity and cesarean delivery (as opposed to natural delivery) were recognized as significant threatening factors in 90% and nearly 75% of the studies, respectively. Furthermore, the results of this review show that mothers attended by skilled birth personnel, under prenatal care, as well as educated women are less likely to die from pregnancy-related causes. Moreover, delay in seeking health services and making decisions for delivery in all the eight observational evidences and factors related to mothers’ health (including the history of underlying disease, medical comorbidities, having complications during pregnancy or postpartum period, and hospitalization during pregnancy) were significant contributors according to most studies. However, in a few studies, some variables such as BMI, race, skin color, and caste were not recognized as associated factors. In contrast, each of the factors of birth weight, substance misuse, nationality, and previous pregnancy history were significant factors in one study, which we refused to include in Table 4. At this level, there is no consensus on the effect of age at marriage, age at first birth, birth weight, and postnatal care coverage. Details about other determinants at this level were described in Table 4.

Determinants at household level show that improved water sources and sanitation systems were the main contributors in most studies. In addition, low family income or wealth, and rural residence were associated factors in nearly 87% and 70% of studies, respectively. Also in this level, access to electricity in all the evaluated studies, and higher levels of husband education were recognized as significant factors in 80% of the observational studies (details in Table 4). Other factors mainly related to the condition of living, including the type of flour and toilet, building material of the house, and type of family were the associated factors, which are not shown in Table 4.

At community or social levels, poor human resources in 73% of the examined studies, and poor accessibility or availability of health facilities (including the number of hospitals, primary health care, hospital beds, and equipment, etc.) in 79% of the 19 examined studies were the main contributors that threaten maternal health. Moreover, health expenditure shares (including higher share of out-of-pocket or private expenditures, and lower share of total health of GDP or public expenditures) and higher total fertility rate per woman were other associated factors in 89% and 77% of the included studies, respectively. In this category, there is no consensus on the effect of quality of services, and less use of contraception (details in Table 4). A few of the selected studies also recognized other factors such as life expectancy, need for emergency obstetric care, total calories consumed, and fat residuals as significant factors, which we refused to enter in Table 4. In terms of economic factors that were mainly studied in ecological studies, lower-income countries levels (GNP or GDP) in 89% of the studies, unemployment rate in all studies, and poverty in 57% of the examined studies were significant threatening factors. However, income inequality and foreign investment are not known as significant factors. In contrast, debt rate, agricultural production indices, and private-sector infrastructure were other contributors in a study, which we refused to enter in Table 4. In addition, results show that maternal mortality was also caused by social, political, and cultural factors. Thus, levels of education and governance (corruption index, instability, voice and accountability, etc.) were the main contributors in five ecological studies. Also, gender inequality and human development index both in four original studies were known as main factors in this category (details in Table 4). Also, other factors such as paved roads, annually published scientific papers, percentage of the indigenous population, and education efficiency were significant factors, which we refused to enter in Table 4.

### Maternal mortality determinants based on income levels of countries

A summary of our findings is shown in Table 4. As it shows, the attributable risk of maternal death is varied based on income levels and development levels of countries; more than 80% of the examined studies and all four ecological studies found poor income levels of countries (GDP/GNP) and human development index strongly associated with maternal death, respectively.

Among the factors at individual levels, prenatal care, delay in decisions, type of delivery, and hospitalization during pregnancy in most studies (except the type of delivery in lower-middle income group) were significant factors in all different income levels. In six out of seven global studies, skilled birth attendance was a significant contributor in low-income, less developed or developing countries. Also, maternal education as another main factor, was an essential contributor in low and lower-middle-income countries, and more than 80% and 70% of the examined studies have shown a significant relationship, respectively. However, there is no consensus on this factor in upper-middle-income countries. Some factors like history of underlying disease, medical comorbidities, high-risk pregnancy, length of labor, and pregnancy stage were recognized as risk factors in low or lower-middle-income countries (Table 4).

Based on this review, most studies statistically accepted that less access to clean water and improved sanitation negatively affected maternal death in low and lower-middle-income countries, respectively. In addition, in poor countries, improvement in the availability or accessibility of health services and health expenditure shares were essential determinants in the reduction of maternal death. Based on our findings, reduction in the share of out-of-pocket and private health expenditures and increase in the share of total health expenditures as a percentage of GDP and public expenditures were most responsible for pregnancy-related deaths, particularly in low-income countries as well as other income groups. Moreover, the risk of pregnancy-related death was highly significant among the population below poverty line in all existing evidence, and the use of contraception in many studies in low-income countries. However, poverty in upper-middle-income countries was not significantly associated with maternal death. Moreover, gender inequality and gender empowerment measures (autonomy, economic empowerment, and attitudes towards violence) in low and lower-middle-income countries were associated contributors in most studies. However, gender empowerment measures did not affect maternal death in two ecological studies, and there is no consensus on the significance of gender inequality in our global studies. Details of the other variables is shown in Table 4.

## Discussion

This study aimed to review and overview maternal mortality determinants in the world and categorize them based on income levels of countries. Our included studies show that most of these studies were conducted ecologically and were case–control studies and 58% of them were carried out in low and lower-middle-income countries.

According to the result of this study, maternal age, gravidity, type of delivery, education of mothers, pregnancy care, skilled birth attendance, and maternal health status are the main factors at individual level. At family level, factors such as access to improved water and sanitation, region of residence, family income or wealth, and other factors related to living conditions were significantly associated with maternal death. However, the systematic review conducted in Iran [156] failed to find the place of living as a risk factor because of having more access to primary health care among rural women in Iran. At community or social level, availability of health services, total fertility rate, health expenditures shares, income level, governance, inequality and education were main contributors.

Our review shows that women aged 18–34 years were less likely to die as compared with age groups of 35–49 or under 18 years. A possible reason for the high risk of maternal death among women aged 35–49 years is due to weakened uterus and anemia, and becoming pregnant is too risky for women older than 35 years and studies showed that the majority of women in this age group were not educated. Studies indicate that most maternal deaths occurred in women with no antenatal care and delay in seeking health services. This may be due to lack of awareness about the seriousness of maternal health. The type of delivery can also affect maternal mortality, as most cases of obstetric hemorrhage and emergency postpartum hysterectomy are associated with CS deliveries [75].

Among original studies, two observational studies in lower-middle income countries [93, 144], and an observational study in the west of Iran [93], as upper middle-income country, demonstrate that skilled birth attendance has no significant relationship with maternal mortality. However, it doesn’t seem that skilled birth attendance has no effects on maternal mortality at this income level as those studies were conducted with a small sample size and limited time span. A longitudinal study in Chile, as a lower middle-income level country, showed this factor as a significant contributor in adjusted models. Some global or regional studies, mainly including less developed or developing countries, accepted this factor [76, 130, 138].

Based on our findings, households’ access to improved water and sanitation especially in low-income countries were negatively associated with maternal death. In line with this, Benova et al. [15] suggested that women in households with poor sanitation were 3.14 times more likely to die compared to women with better sanitation. The concept is that poor access to sanitation and water can provide the conditions for the prevalence of infectious diseases, which can directly affect maternal death. Studies also show that access to improved sanitation is associated with income level of countries [79, 115]; a study shows that clean water and sanitation access improved by 14% due to economic growth in low and middle-income countries from 1990 to 2010 [73]. Based on WHO data [157], access to improved sanitation and water was always over 98% among high-income countries since 1990, while in low-income countries, access to clean water fluctuated around 46% to 66%, and access to sanitation fluctuated around 13% to 28% from 1990 to 2015.

Governance is one of the key factors that affects maternal mortality. Governance can be described as a set of traditions and conventions that determines the practice of authority in a particular country. It comprises not only the processes through which governments are selected, held accountable, monitored, and replaced, but also the capacity of governments to efficiently manage resources and formulate, implement, and enforce appropriate policies and regulations. In addition, governance regulates the level of respect received by the citizens and the state for conventions and laws that govern the economic and social interactions in the community. Through better governance, public spending can effectively and efficiently enhance health and education outcomes.

Some economic and health system-related factors such as the Gross National Income per capita and per capita government expenditure on health showed an inverse correlation with MMR. In contrast, private sector and out-of-pocket health expenditure showed a significant direct correlation with MMR: the more private sectors and out-of-pocket health expenditures in a country, the higher the MMR. Since appropriate government financing can ensure better access to some essential maternal health services, greater levels of health expenditure will be required for developing countries to achieve MDG on maternal mortality. Between 1995 and 2014, the average total health expenditure (%GDP) varied from 5% in low-middle income countries, 48% of which was paid by public expenditure, to nearly 11% in high-income countries, 61% of which was paid by public expenditure. Based on WHO statistics, the average out-of-pocket share (% total health expenditure) varied from nearly 41% in low-middle income countries to nearly 15% in high-income countries in the same period [158].

According to the literature, poverty positively affects maternal mortality as an economic factor, especially in the low-income countries. According to the evidence, poverty is linked to maternal mortality through malnutrition [99]. Malnutrition has been associated with anemia that is one of the main causes of maternal death [159]. Malnutrition may lead to chronic iron deficiency and anemia, which can make women prone to hemorrhage and infections [160]. Furthermore, women who experience malnutrition early in life are usually smaller, which increases the likelihood of obstructed labor [160].

Also, low human development index (HDI) in all evaluated studies was recognized as a significant contributing factor. HDI is one of the powerful indexes, which is a geometric means with three dimensions of life expectancy at birth, education, and GDP per capita. Much evidence shows that MMR reflects the general health status of a country and its development [130].

Moreover, education as a social factor is the other main contributor. As seen in Table 4, among 20 studies that examined the effects of education on maternal mortality at community level, only one did not confirm this. This shows that education is influential in reducing maternal mortality. Educated women are more exposed to having an informed reproductive behavior, and they access reproductive health facilities frequently and timely. When women are educated, they increasingly improve their status, gain autonomy, awareness, responsibility, and control their fertility and reproductive activities such as in the use of contraceptives, prenatal and postnatal care, and health facility delivery [13]. Literature also shows that education in low and lower-middle-income countries is essential, which may have happened due to the low level of social and economic infrastructures in them.

Other factors including households’ income, employment, urbanization, inequalities, gender empowerment, human resource, availability of services were the other main factors particularly in less developed or developing countries (Table 4).

To the best of our knowledge, this is the first systematic review to present a robust summary of individual and ecological determinants of maternal mortality in the world based on income level of the countries. We used a sensitive search strategy in different sources, and finally identified 121 studies (109 original studies and 12 review articles). However, this review has several potential limitations. As the main limitation of this study, we only included studies in English with access to full-text documents. To compensate this, we did a rigorous search on different electronic databases, journals, and paper references, and the abstracts of excluded studies were reviewed to reduce bias. As with any systematic review, we may have missed some studies due to relatively a short list of search terms. Also, some studies did not provide access to the full text and thus they had to be excluded from this review. However, we minimized this by exhausting the search process through key terms, and the search strategy employed multiple academic and grey literature databases. As another limitation, all included studies had analytical observational and review design, so the cause-effect relation between determinants and maternal mortality could not be established. The results of this review can help researchers to understand the main determinants of maternal mortality based on income level of the countries and provide an appropriate space for research. This study can also provide comprehensive views for policymakers to reduce maternal mortality.

## Conclusions

This review aims to comprehensively show the determinants of maternal mortality. The results of this study demonstrate the individual-level factors (e.g., age and parity), household-level factors such as region of residence, access to improved water etc., and community-level factors such as socio-economic, cultural, and health care system factors associated with maternal mortality. Based on extracted determinants, providing health services by making them affordable and available, access to quality health care, access to prenatal care, family planning care, and emergency obstetric care, increasing education levels, improving the condition of living and infrastructures, and the use of contraception can play an important role in the reduction of maternal mortality. Further studies are recommended to reach a consensus regarding certain determinants such as age at marriage, postnatal care coverage, etc.

## Data Availability

All data generated or analyzed during this study are included in this published article.
